# Supplementary material for: Chirality-dependent electrical transport properties of carbon nanotubes obtained by experimental measurement
Source: Nat Commun. 2023 Mar 25;14:1672. doi: 10.1038/s41467-023-37443-7 (PMC10039901; doi:10.1038/s41467-023-37443-7)
Supplement: Supplementary file 1 — Supplementary Inframtion [file 41467_2023_37443_MOESM1_ESM.pdf]

Supplementary Information

Chirality-Dependent Electrical Transport Properties of Carbon Nanotubes Obtained by Experimental Measurement

Wei Su, [1,2,3,6] Xiao Li, [1,2,3,6] Linhai Li, [1,2,3] Dehua Yang, [1,3] Futian Wang, [1,2,3] Xiaojun Wei,[1,2,3,4] Weiya Zhou, [1,2,3,4] Hiromichi Kataura,[5] Sishen Xie, [1,2,3,4] and Huaping Liu[1,2,3,4,*]

[1]*Beijing National Laboratory for Condensed Matter Physics, Institute of Physics, Chinese Academy of Sciences, Beijing 100190, China*

[2]*Center of Materials Science and Optoelectronics Engineering, and School of Physical Sciences, University of Chinese Academy of Sciences, Beijing 100049, China*

[3]*Beijing Key Laboratory for Advanced Functional Materials and Structure Research, Beijing 100190, China*

[4]*Songshan Lake Materials Laboratory, Dongguan, Guangdong 523808, China*

[5]*Nanomaterials Research Institute, National Institute of Advanced Industrial Science and Technology (AIST), Tsukuba 305-8565, Japan.*

[6]*These authors contributed equally: Wei Su, Xiao Li*

Corresponding author: liuhuaping@iphy.ac.cn

Supplementary Figure 1. Purity evaluation of the single-chirality SWCNTs used in the present work.
Supplementary Figure 2. Raman spectra characterization of single-chirality SWCNTs.
Supplementary Figure 3. Histograms of length distribution of 11 kinds of single-chirality SWCNTs with different diameter and family.
Supplementary Figure 4. The performances of the TFTs constructed by low-density (6, 5) SWCNT films.
Supplementary Figure 5. AFM images of the 11 kinds of single-chirality SWCNT films.
Supplementary Figure 6. The calculated percolation threshold of different (*n, m*) SWCNTs based on their average lengths.
Supplementary Figure 7. Height characterization of the nanotubes in the networks by AFM.
Supplementary Figure 8. A typical TEM image of a single SWCNT from a deposited film.
Supplementary Figure 9. Output characteristic curves of *p/n* branches of a typical (9, 2) SWCNT TFT.
Supplementary Figure 10. The optical absorption spectra of the DOC-dispersed (6, 5) SWCNTs before and after displacing with SDS surfactant.
Supplementary Figure 11. The performances of the TFTs constructed by the SDS-dispersed SWCNTs.
Supplementary Figure S12. A typical YFM curve calculated from transfer curve of a TFT.
Supplementary Figure 13. The relationship of threshold voltage and hysteresis with chirality, family, Type and diameter.
Supplementary Figure 14. DOS distribution of SWCNTs.
Supplementary Figure 15. The relationship between the channel resistance and the relative conductance.
Supplementary Note 1: The calculation of the percolation threshold.
Supplementary Note 2: The displacement of the DOC-dispersed (6, 5) SWCNTs with SDS
Supplementary Note 3: Methods to extract threshold voltage, on-current, mobility, contact resistance ($2R_c$) and channel resistance (R_L).
Supplementary Table 1 The calculated percolation threshold.

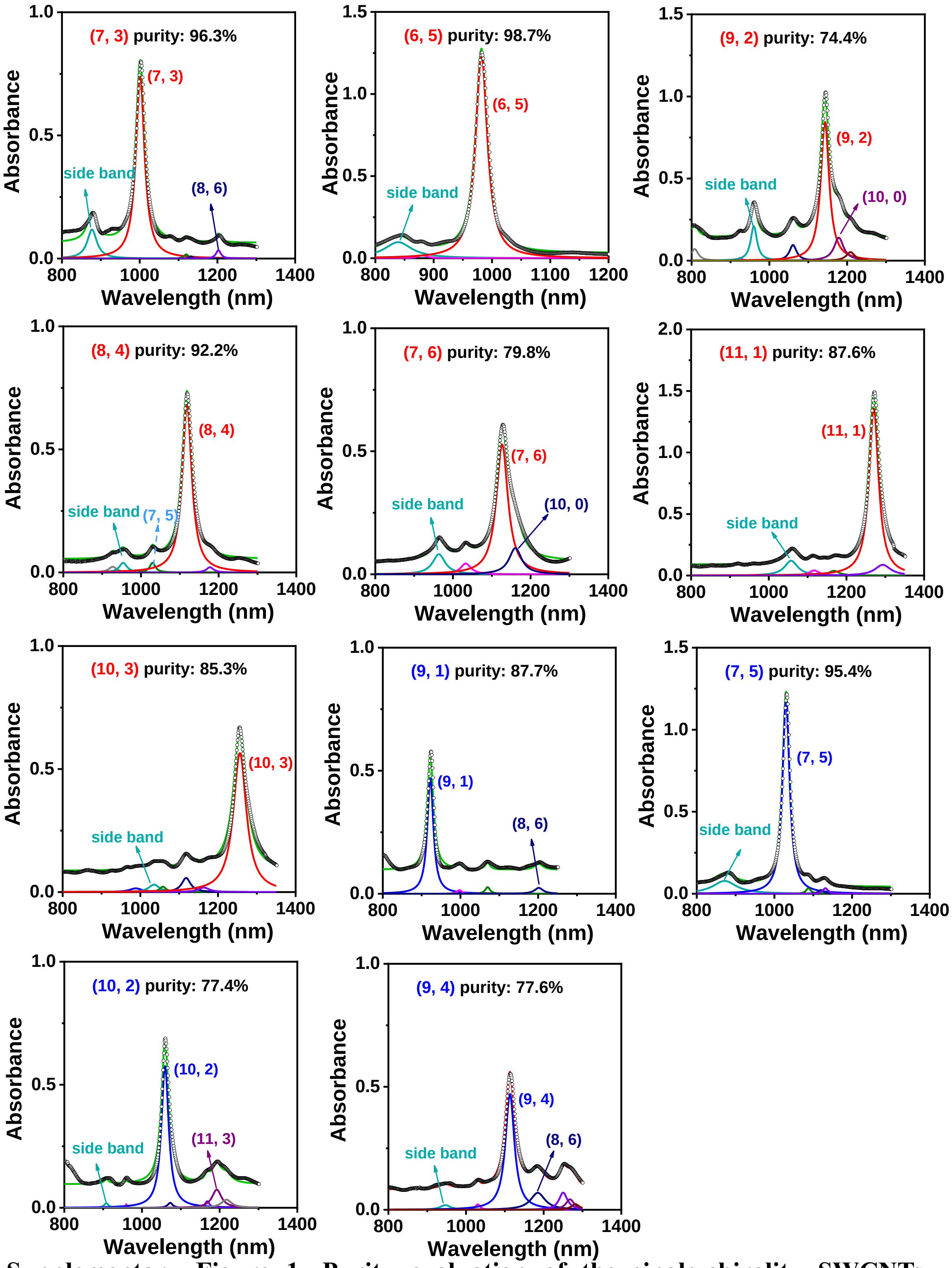

Supplementary Figure 1. Purity evaluation of the single-chirality SWCNTs employed in the present work. PeakFit software was used to simulate the near-infrared spectra of the individual (*n, m*) species with wavelengths from 800 to 1350 nm.[1] The purity of each (*n, m*) fraction was computed as the ratio of the area of the dominant peak to the sum of all peak areas.

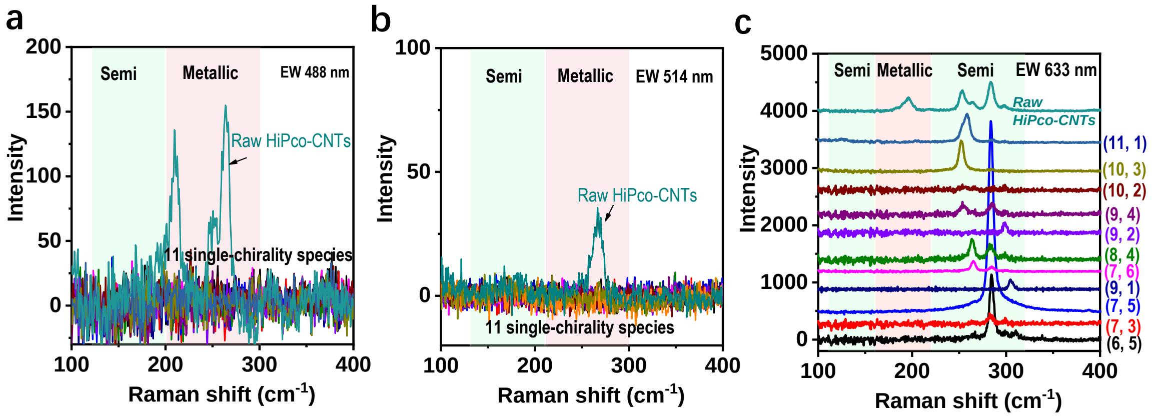

Supplementary Figure 2. Raman spectra characterization of single-chirality SWCNTs. Raman spectra of the single-chirality SWCNTs used in the present work with excitation wavelengths of (a) 488nm, (b) 514 nm and (c) 633 nm, respectively. As a comparison, Raman spectra of the Raw HiPco-SWCNTs were also measured. The results show that no metallic SWCNTs were detected in the single-chirality species.

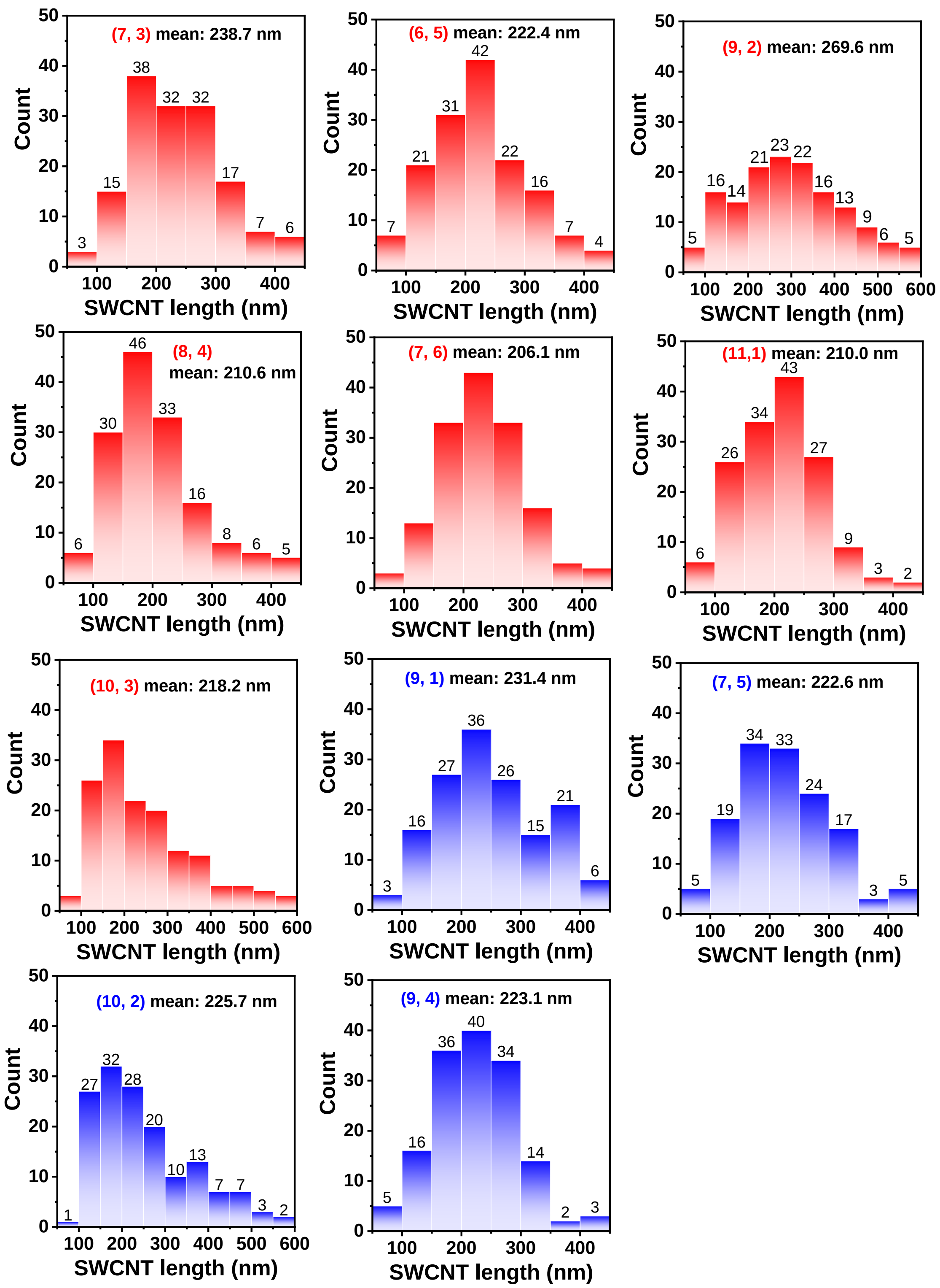

Supplementary Figure 3. Histograms of length distribution of 11 kinds of single-chirality SWCNTs with different diameter and family. In order to obtain sparse SWCNT films, we take substrates immersing into SWCNT solutions for just 1 minute while dense film for 1 hour. The type of SWCNT is distinguished by color where the blue is for Type I and red for Type II SWCNT.

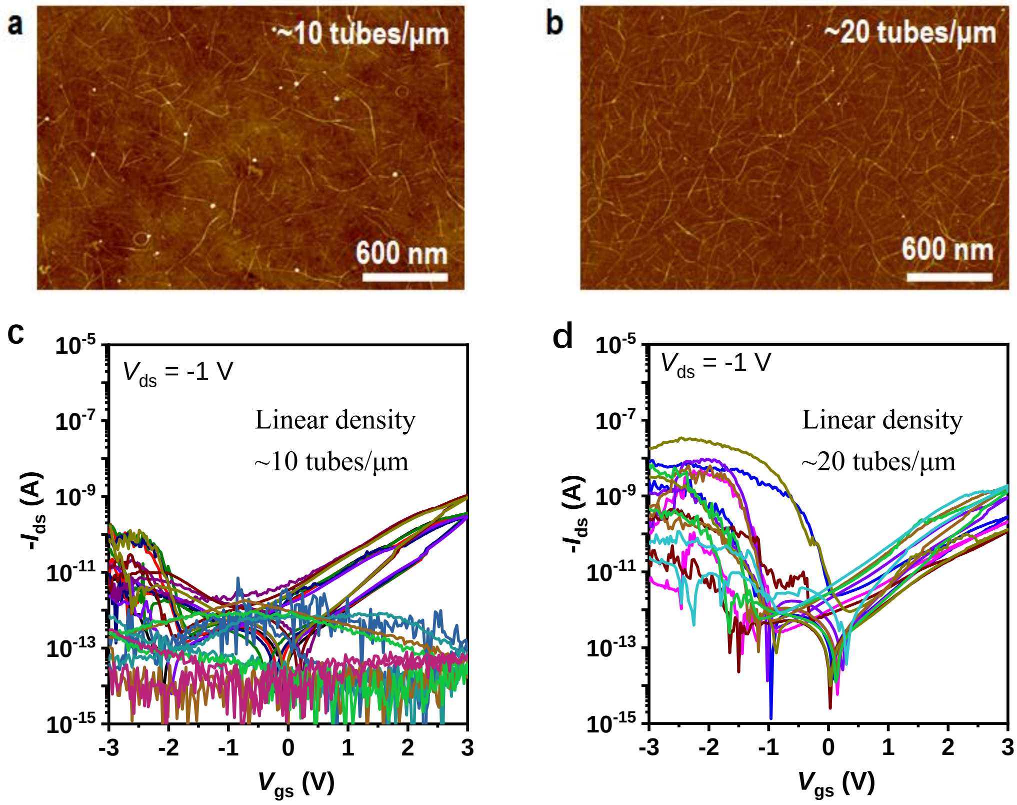

Supplementary Figure 4. The performances of the TFTs constructed by low-density (6, 5) SWCNT films. (a) and (b) show the AFM images of (6,5) SWCNT films with linear density of ~10 and ~20 tubes/um. (c) and (d) is the corresponding transfer curves of the TFTs. The detailed fabrication process of TFTs is described in the method part in the main text. The fabricated transistors are top-gate structures with channel length and width of 2 and 20 μm, respectively, in which 15-nm-thick HfO_2 was used as a dielectric layer.

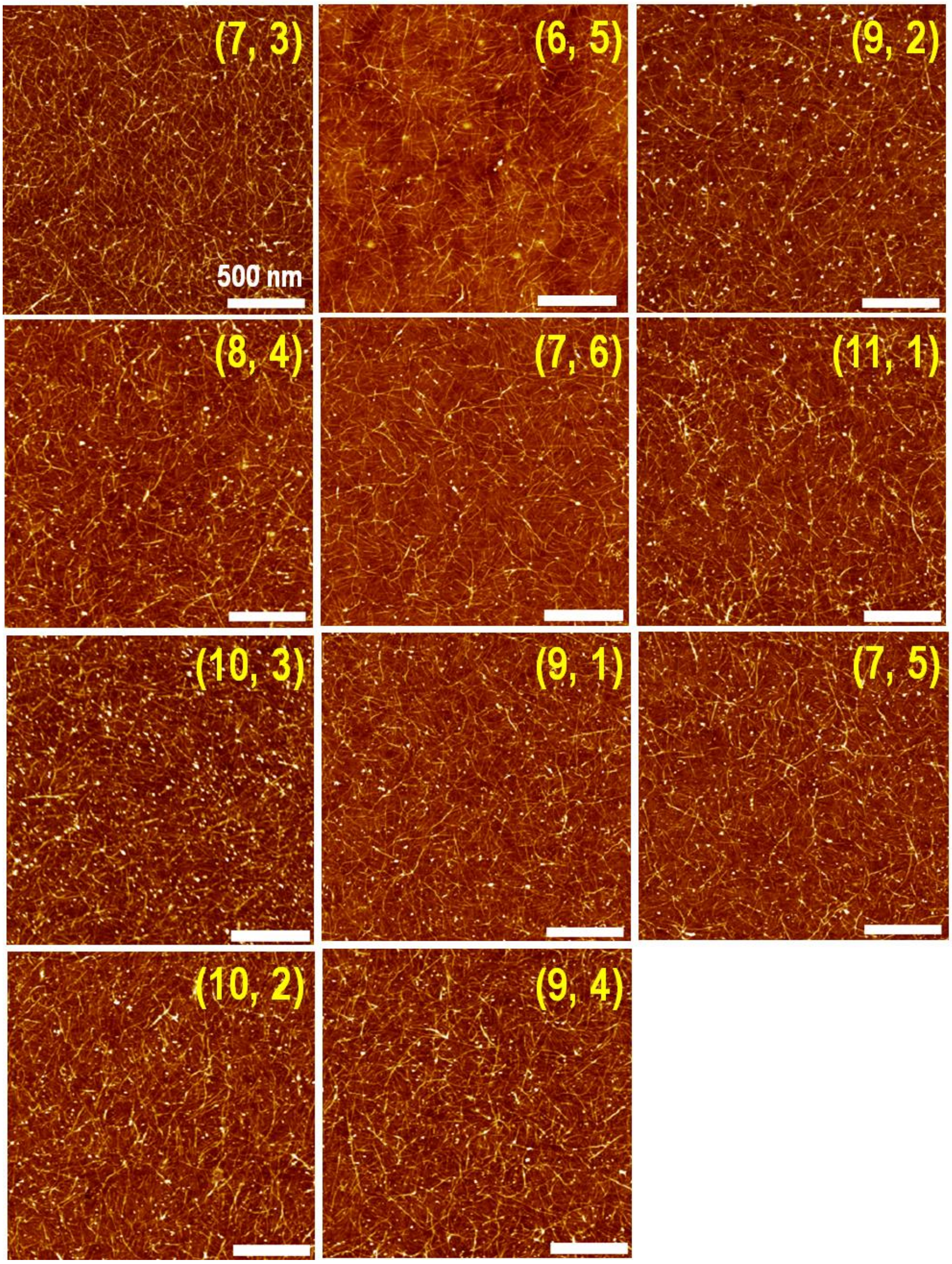

Supplementary Figure 5. AFM images of the 11 kinds of single-chirality SWCNT films. The functionalized SiO_2/Si substrates by PLL were immersed into the as-prepared single-chirality SWCNT solution, whose pH were controlled by addition of $NaHCO_3$. The linear density of each film is 32±5 tubes/μm.

Supplementary Note 1: The calculation of the percolation threshold.

The percolation threshold of each kind of (*n, m*) SWCNT film was calculated based on the following equation:[2, 3]

$$N_{th} = \frac{1}{\pi}\left(\frac{4.236}{L_{cnt}}\right)^2$$

Where N_{th} is the percolation threshold of (*n, m*) SWCNTs, L_{cnt} represents mean length of (*n, m*) SWCNTs. The results show that the percolation thresholds of (*n, m*) SWCNTs is about 10 tubes/um (as shown in Table S1 and Figure S6), which is much lower than the average linear density of the SWCNT films (~32 tubes/um) used to construct transistors in our present work (Figure 1g). Therefore, small fluctuation in the average densities of different chiral SWCNTs would not change the difference in TFT behaviors.

Supplementary Table 1 The calculated percolation threshold.

**(*n, m*)**	**(7, 3)**	**(6, 5)**	**(9, 2)**	**(8, 4)**	**(7, 6)**	**(11, 1)**	**(10, 3)**	**(9, 1)**	**(7, 5)**	**(10, 2)**	**(9, 4)**
Length (nm)	238.70	222.40	269.60	210.60	206.10	210.00	218.20	231.40	222.60	225.70	223.10
Area Density (tubes/um^2)	100.24	115.48	78.58	128.78	134.46	129.52	119.96	106.67	115.27	112.12	114.75
Linear density (tubes/um)	10.01	10.75	8.86	11.35	11.60	11.38	10.95	10.33	10.74	10.59	10.71

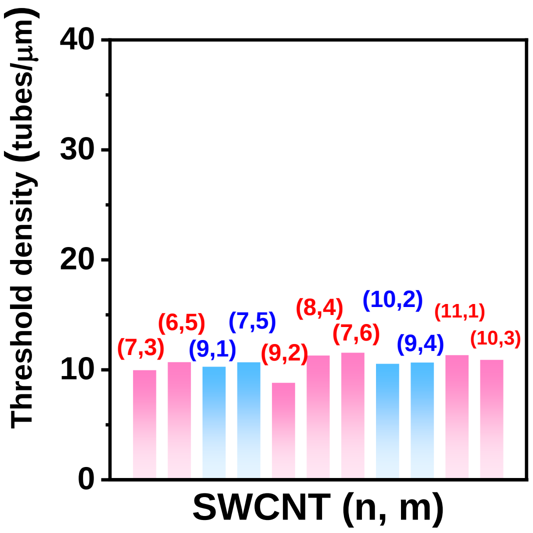

Supplementary Figure 6. The calculated percolation threshold of different (*n, m*) SWCNTs based on their average lengths.

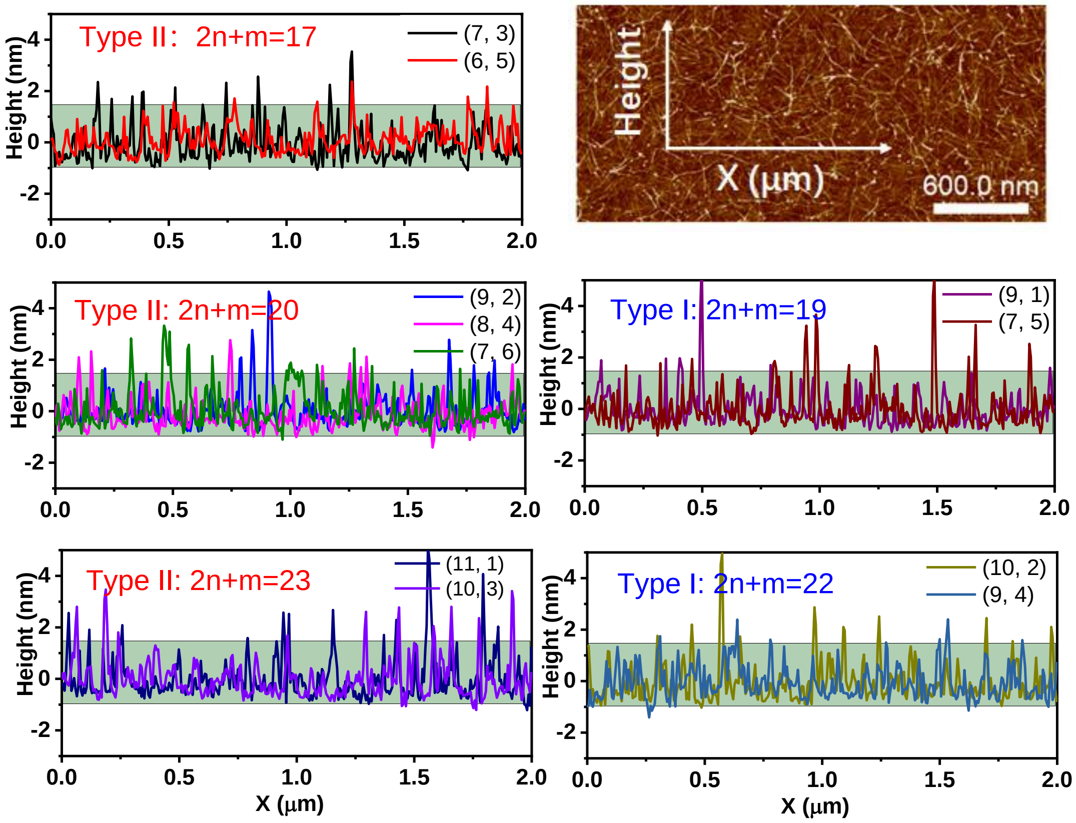

Supplementary Figure 7. Height characterization of the nanotubes in the networks by AFM. The (*n*, *m*) SWCNT films with an average density of ~32 tubes/um show high density uniformity.

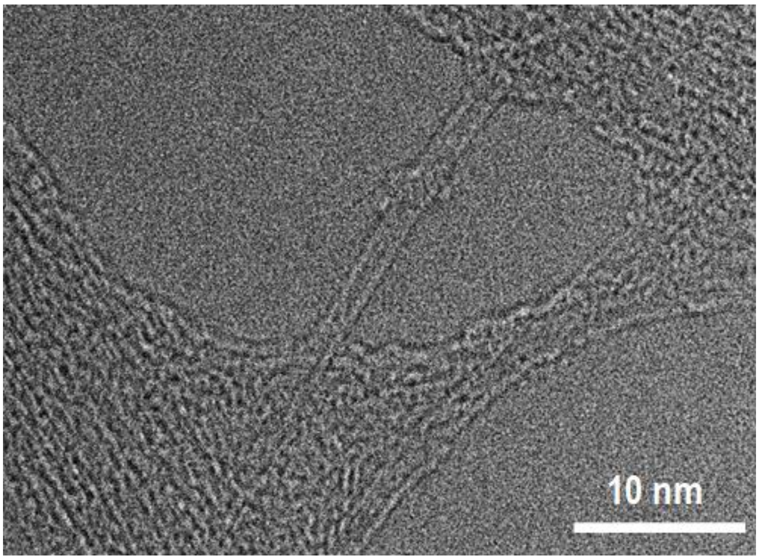

Supplementary Figure 8. A typical TEM image of a single SWCNT from a deposited film. Before deposition, the SWCNTs were dispersed in a DOC solution with a concentration of 0.05%. After film deposition, the surfactant molecules were removed by repeated cleaning with deionized water. The results show that a few DOC molecules remained on SWCNTs.

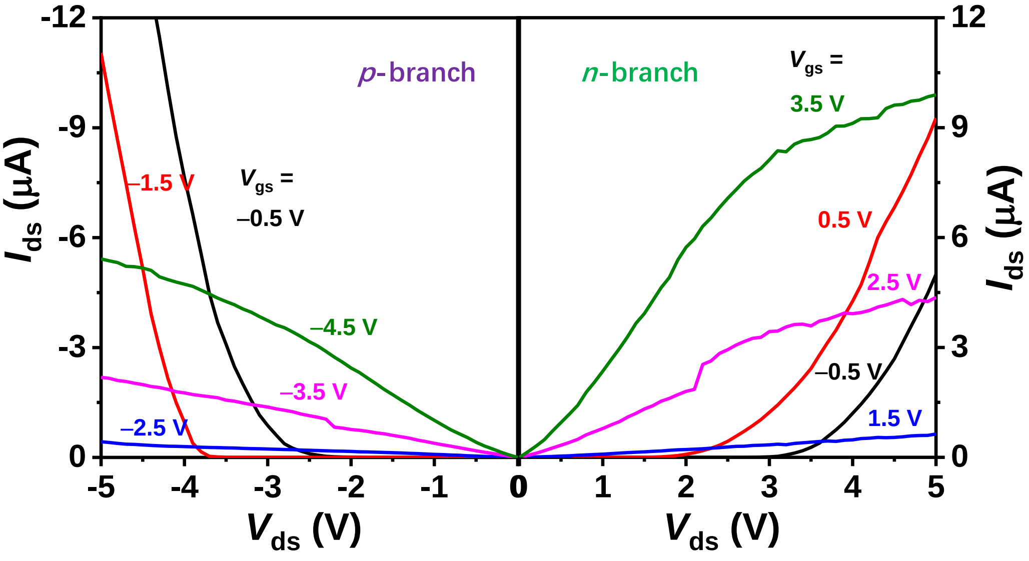

Supplementary Figure 9. **Output characteristic curves of *p/n* branches of a typical (9, 2) SWCNT TFT**. Two branches demonstrate the similar gate-controlling output characteristics.

Supplementary Note 2: The displacement of the DOC-dispersed (6, 5) SWCNTs with SDS

The optical absorption spectra of the DOC and SDS-dispersed SWCNTs are different due to different dielectric environment. To verify whether DOC can be displaced by SDS, we characterized the optical absorption spectra of the DOC-dispersed (6, 5) SWCNTs before and after being displaced by SDS. As shown in Figure S10, the S_{11} optical absorption peak of the DOC-dispersed (6, 5) SWCNTs exhibit a blue-shift of 4 nm after replacement with SDS, which coincided with that of the SDS-dispersed (6, 5) SWCNTs (black curve), sufficiently indicating that the DOC coating was replaced by SDS.

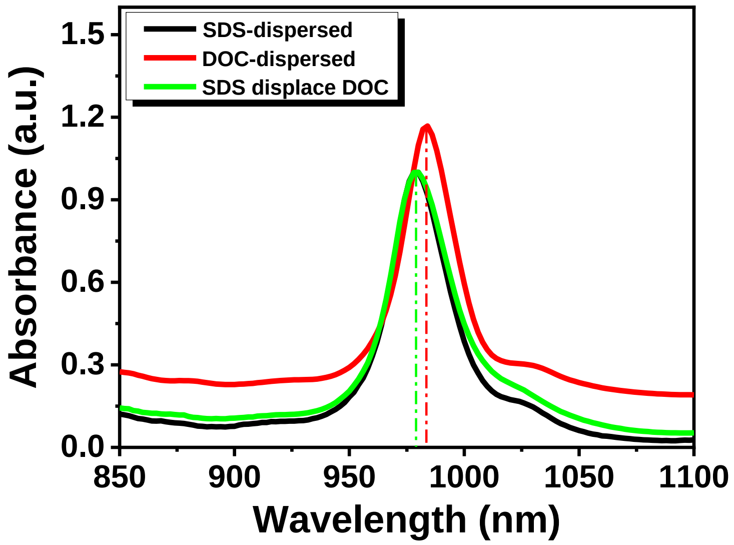

Supplementary Figure 10. The optical absorption spectra of the DOC-dispersed (6, 5) SWCNTs before and after displacing with SDS surfactant. After displacing with SDS, the optical absorption spectrum exhibits a blue shift of 4 nm, which coincide with that of the SDS-dispersed (6, 5) SWCNTs. The results indicate that the DOC coating around SWCNTs could be displaced with SDS. For comparison, the optical absorption spectrum of the DOC dispersed (6,5) SWCNTs is upshifted vertically.

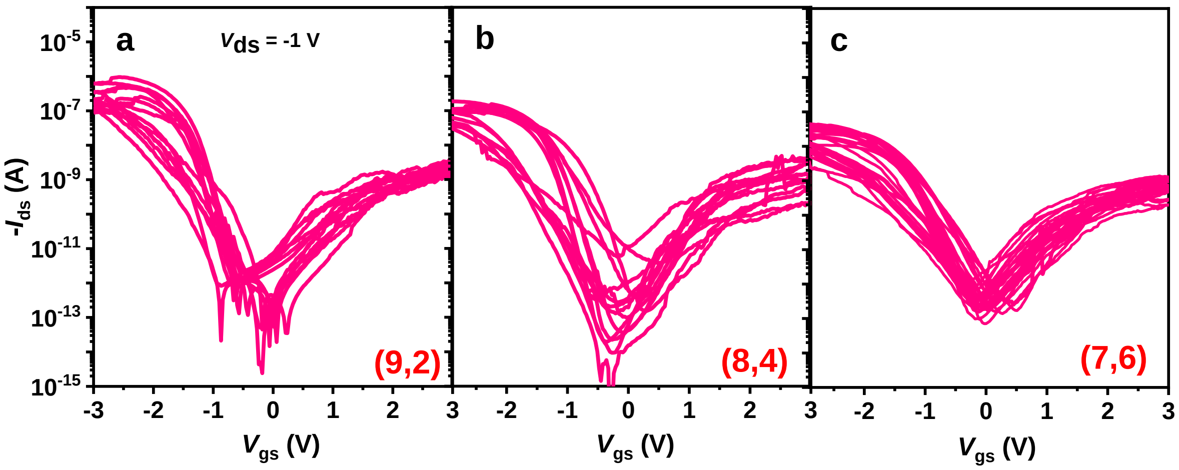

Supplementary Figure 11. **The performances of the TFTs constructed by the SDS-dispersed SWCNTs.** The transfer curves of the TFTs constructed by Type II SWCNTs films of (a) (7, 6), (b) (8, 4) and (c) (9, 2) SWCNTs, which were deposited by SDS-dispersed solutions. The on-state current of the TFTs shows the same trend as that of the DOC-dispersed SWCNTs. These results further confirm that the measured chirality-dependent electrical properties are not dominated by the selective interaction of DOC with SWCNTs.

Supplementary Note 3: Methods to extract threshold voltage, on-current, mobility, contact resistance (2R_c) and channel resistance (R_L).

(1) Threshold voltage (V_{th}). The V_{th} is obtained from YFM curve where Y is defined as:[4,5]

$$Y = \frac{I_{ds}}{\sqrt{g_m}}$$

where g_m is the transconductance. In the YFM curve, the intersection of the extension line of linear segment intersects with V_{gs} axis is V_{th}.

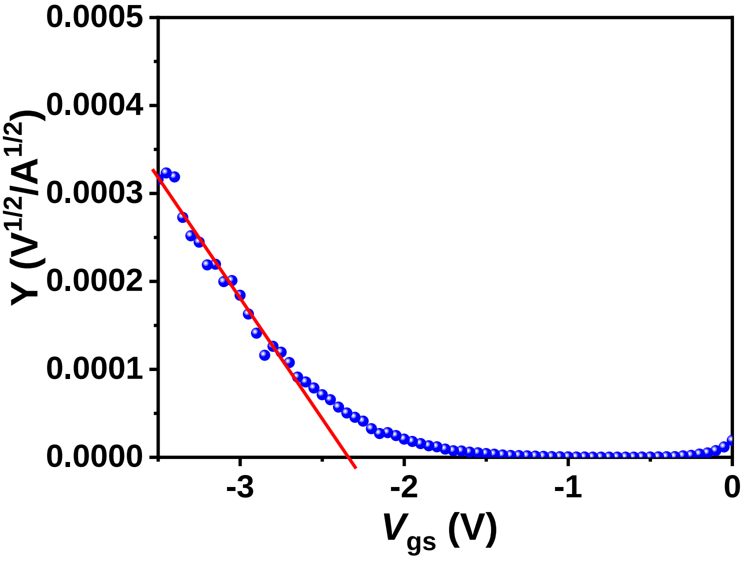

Supplementary Figure 12. A typical YFM curve calculated from transfer curve of a TFT.

(2) On-current. On-current is defined as the current at $V_{gs} = V_{th}$ ±1 V (+ for *n*-branch and – for *p*-branch) in the transfer characteristics of I_{ds}-V_{gs}.[4]

(3) Hysteresis. Hysteresis is defined as the difference of the gate voltage at $I_{ds} = 1$ nA in forward and reverse scanning transfer curves.

(4) Mobility, 2R_c and R_L calculation. The total resistance of TFT can be expressed as

$$R_{tot} = \frac{V_{ds}}{I_{ds}} = 2R_c + R_L$$

where R_L can be approximated as[6]:

$$R_L = \frac{L}{\mu * W * C_{ox}(V_{gs} - V_{th})}$$

Therefore,

$$\frac{V_{\mathrm{ds}}}{I_{\mathrm{ds}}} = 2R_{\mathrm{c}} + R_{\mathrm{L}} = \frac{L}{\mu * W * C_{\mathrm{ox}}(V_{\mathrm{gs}} - V_{\mathrm{th}})} + 2R_c$$

$$\text{and } I_{\mathrm{ds}} = \frac{V_{\mathrm{ds}} * C_{\mathrm{ox}} * u * W * (V_{\mathrm{gs}} - V_{\mathrm{th}})}{L + 2R_{\mathrm{c}} * u * W * C_{\mathrm{ox}}(V_{\mathrm{gs}} - V_{\mathrm{th}})}$$

g_m was calculated as:

$$g_m = \frac{dI_{\mathrm{ds}}}{dV_{\mathrm{gs}}} = \frac{V_{\mathrm{ds}} * C_{\mathrm{ox}} * u * W/L}{\left[1 + 2R_{\mathrm{c}} * W * u * \frac{C_{\mathrm{ox}}}{L} * (V_{\mathrm{gs}} - V_{\mathrm{th}})\right]^2}$$

In YFM, Y function is defined as

$$Y = \frac{I_{\mathrm{ds}}}{\sqrt{g_m}} = \sqrt{\frac{W}{L}\mu C_{\mathrm{ox}} V_{\mathrm{ds}}}(V_{\mathrm{gs}} - V_{\mathrm{th}})$$

as shown in **Figure S12**. If we define the slope of linear segment of YFM curve as a parameter α, then

$$\alpha = \sqrt{\frac{W}{L}\mu C_{\mathrm{ox}} V_{\mathrm{ds}}}$$

So, mobility can be described as

$$u = \frac{L}{V_{\mathrm{ds}} C_{\mathrm{ox}}} * \frac{\alpha^2}{W}$$

where L and W are channel length and width respectively, V_{gs} and V_{ds} are the gate voltage and drain voltage respectively, and C_{ox} is the gate capacitance. In our calculation, L, W, V_{ds} are 2 μm, 20 μm, and −1 V respectively. The gate capacitance C_{ox} per unit area is calculated by the following equation, which considers the electrostatic coupling between SWCNTs:

$$C_{\mathrm{ox}} = \left\{ C_{\mathrm{Q}}{}^{-1} + \frac{1}{2\pi\varepsilon_0\varepsilon_{\mathrm{ox}}} ln\left[\frac{\Lambda_0}{R}\frac{sinh(2\pi t_{\mathrm{ox}}/\Lambda_0)}{\pi}\right]\right\}^{-1} \Lambda_0{}^{-1}$$

where $C_{\mathrm{Q}} = 4.0 \times 10^{-10}\ F/m$ is the quantum capacitance of nanotubes, $\varepsilon_0\varepsilon_{\mathrm{ox}} = 25 \times 8.85 \times 10^{-14}\ F/cm$ is the dielectric constant at the interface between nanotubes film and HfO_2, R is the radius of the corresponding (*n, m*) SWCNT, t_{ox} is the thickness of HfO_2 layer which is 15 nm, and $\Lambda_0{}^{-1}$ is the linear density of SWCNT film which has been shown in **Figure 1** in main text.

And, R_L can be described as

$$R_{\mathrm{L}} = \frac{V_{\mathrm{ds}}}{\alpha^2 (V_{\mathrm{gs}} - V_{\mathrm{th}})}$$

Meanwhile, $2R_{\mathrm{c}}$ can be described as

$$2R_{\mathrm{c}} = \frac{V_{\mathrm{ds}}}{I_{\mathrm{ds}}} - \frac{V_{\mathrm{ds}}}{\alpha^2 \left(V_{\mathrm{gs}} - V_{\mathrm{th}}\right)}$$

where α and V_{th} are both obtained from linear segment of YFM curve.

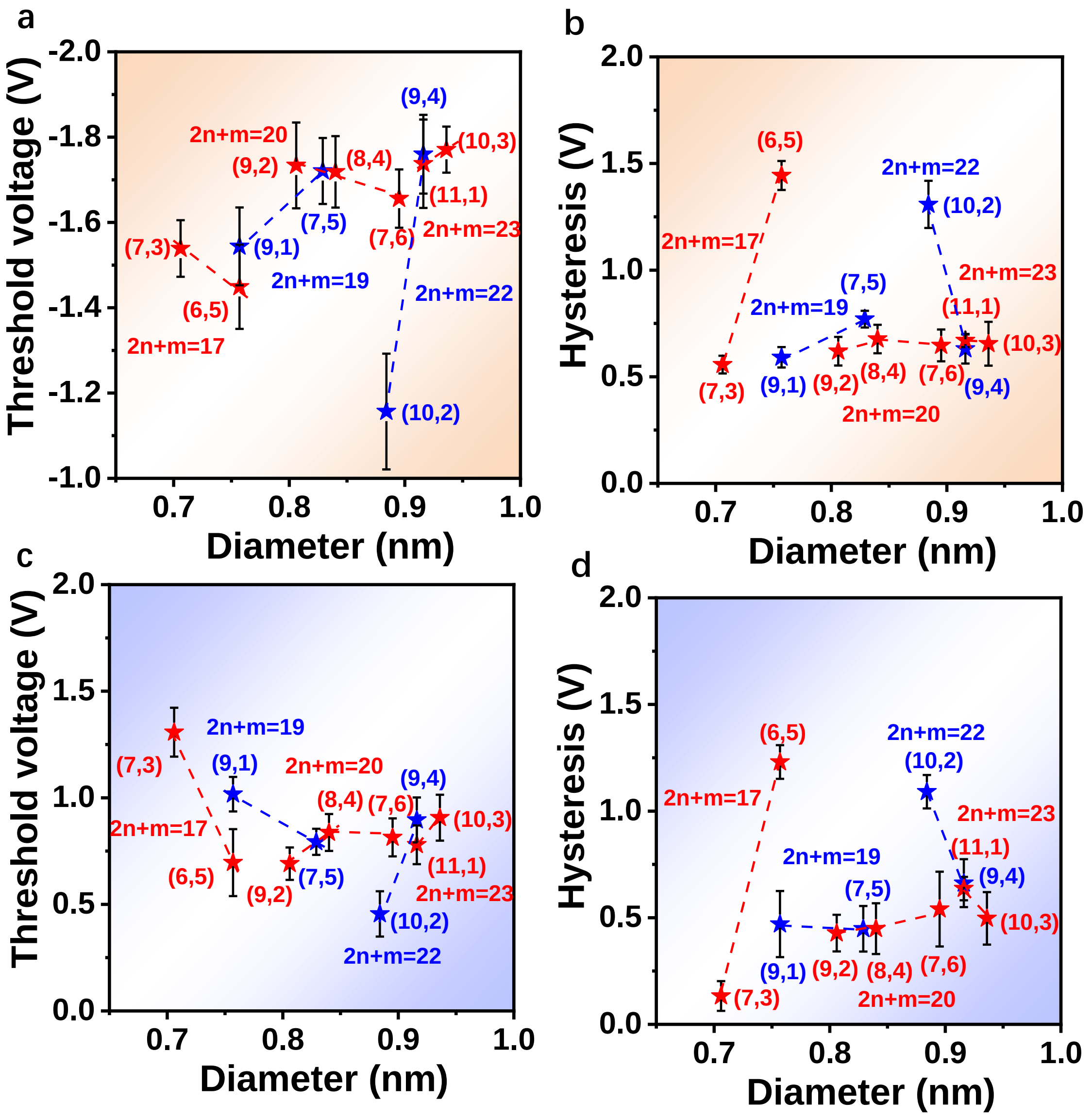

Supplementary Figure 13. The relationship of threshold voltage and hysteresis with chirality, family, Type and diameter. (a-b) Threshold voltage and hysteresis for *p*-branch. (c-d) Threshold voltage and hysteresis for *n*-branch. The results show there is no obvious type, family or chiral angle dependent relationship for both threshold voltage and hysteresis. However, it is notable that the hysteresis of (6, 5) and (10, 2) SWCNTs is clearly larger than other species, which is likely derived from their oxygen-doping defects caused by the oxide dielectric layer (HfO_x). Error bars are the standard deviation of statistics.

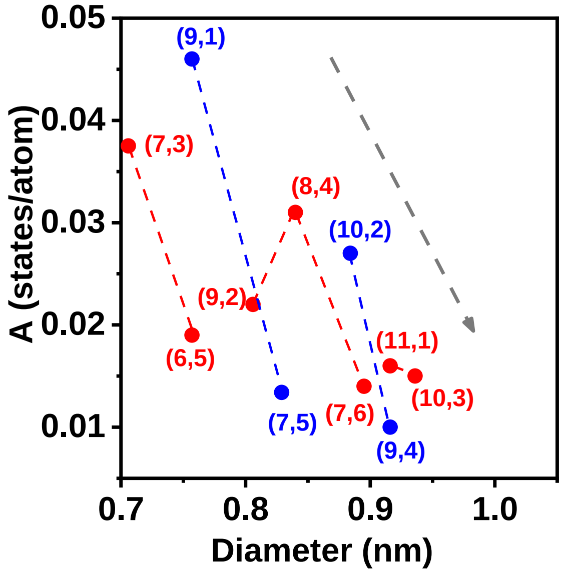

Supplementary Figure 14. **DOS distribution of SWCNTs.** The area of the first conduction band of 11 distinct kinds of SWCNTs as function of their diameter. The data of this figure is from the URL http://www.stat.phys.titech.ac.jp/saito/optCNTs /OptCNT_LDA_DOS_Kato.html and Supplementary reference 7.

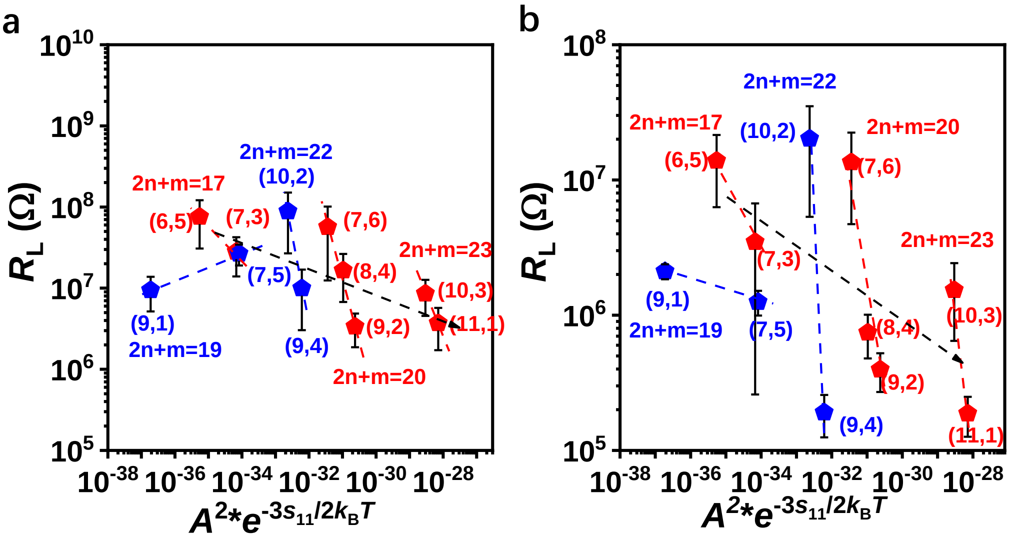

Supplementary Figure 15. The relationship between the channel resistance and the relative conductance. (a) *p*-branch. (b) *n*-branch. The relationship between the channel resistance and the relative junction conductance $A^2*\exp(-3S_{11}/2k_BT)$. The results show that the channel resistance of the same-family SWCNTs decreases with increasing the relative junction conductance. Error bars in Figure are the standard deviation of statistics.

Supplementary References:

1. Liu, H.; Nishide, D.; Tanaka, T.; Kataura, H., Large-scale single-chirality separation of single-wall carbon nanotubes by simple gel chromatography. *Nat. Commun.* 2011, 2, 309.
2. L. Hu, D. S. H., G. Gruner, Percolation in Transparent and Conducting Carbon Nanotube Networks. *Nano Lett.* 2004, 4 (12), 2513-2517.
3. Zhang, J.; Gui, H.; Liu, B.; Liu, J.; Zhou, C., Comparative study of gel-based separated arcdischarge, HiPco, and CoMoCAT carbon nanotubes for macroelectronic applications. *Nano Res*. 2013, 6 (12), 906-920.
4. Cao, Q.; Han, S.; Tulevski, G. S.; Franklin, A. D.; Haensch, W., Evaluation of field-effect mobility and contact resistance of transistors that use solution-processed single-walled carbon nanotubes. *ACS Nano* 2012, 6, 6471-6477.
5. Chang, H.-Y.; Zhu, W.; Akinwande, D., On the mobility and contact resistance evaluation for transistors based on MoS_2 or two-dimensional semiconducting atomic crystals. *Appl. Phys. Lett.* 2014, 104, 113504.
6. Avouris, P. Molecular Electronics with Carbon Nanotubes. *Acc. Chem. Res.* 2002, 35, 1026–1034.
7. Kato, K.; Koretsune, T.; Saito, S., Energetics and electronic properties of twisted single-walled carbon nanotubes. *Phys. Rev. B* 2012, 85, 115448.
