# Supplementary figures and images for: Chirality-dependent electrical transport properties of carbon nanotubes obtained by experimental measurement
Source: Nat Commun. 2023 Mar 25;14:1672. doi: 10.1038/s41467-023-37443-7 (PMC10039901; doi:10.1038/s41467-023-37443-7)

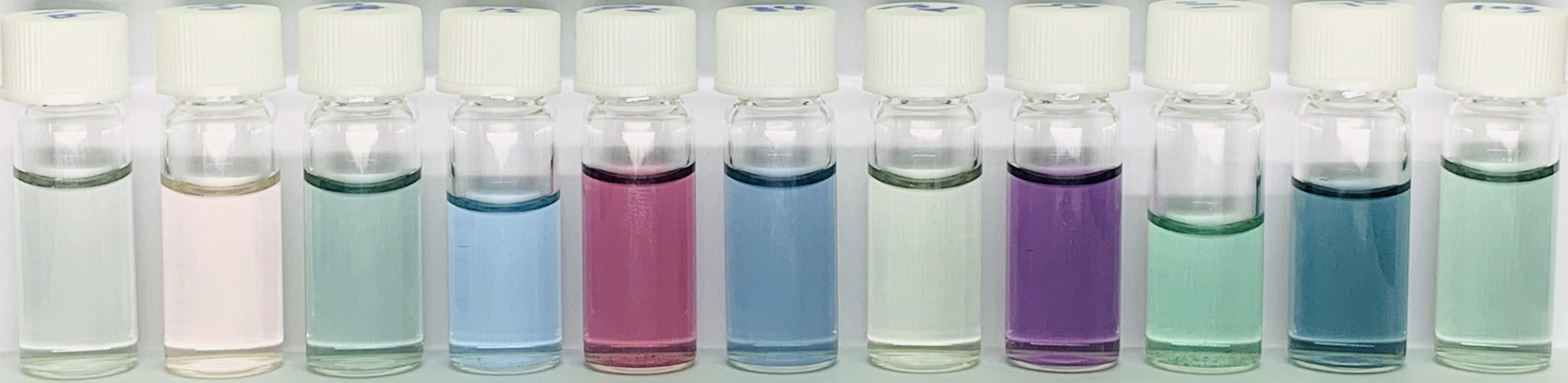

Supplement: Supplementary file 3 — Source Data [file 41467_2023_37443_MOESM3_ESM.zip › 7. Source Data/Figure 1/1b/all SWCNT solutions photo.jpg]

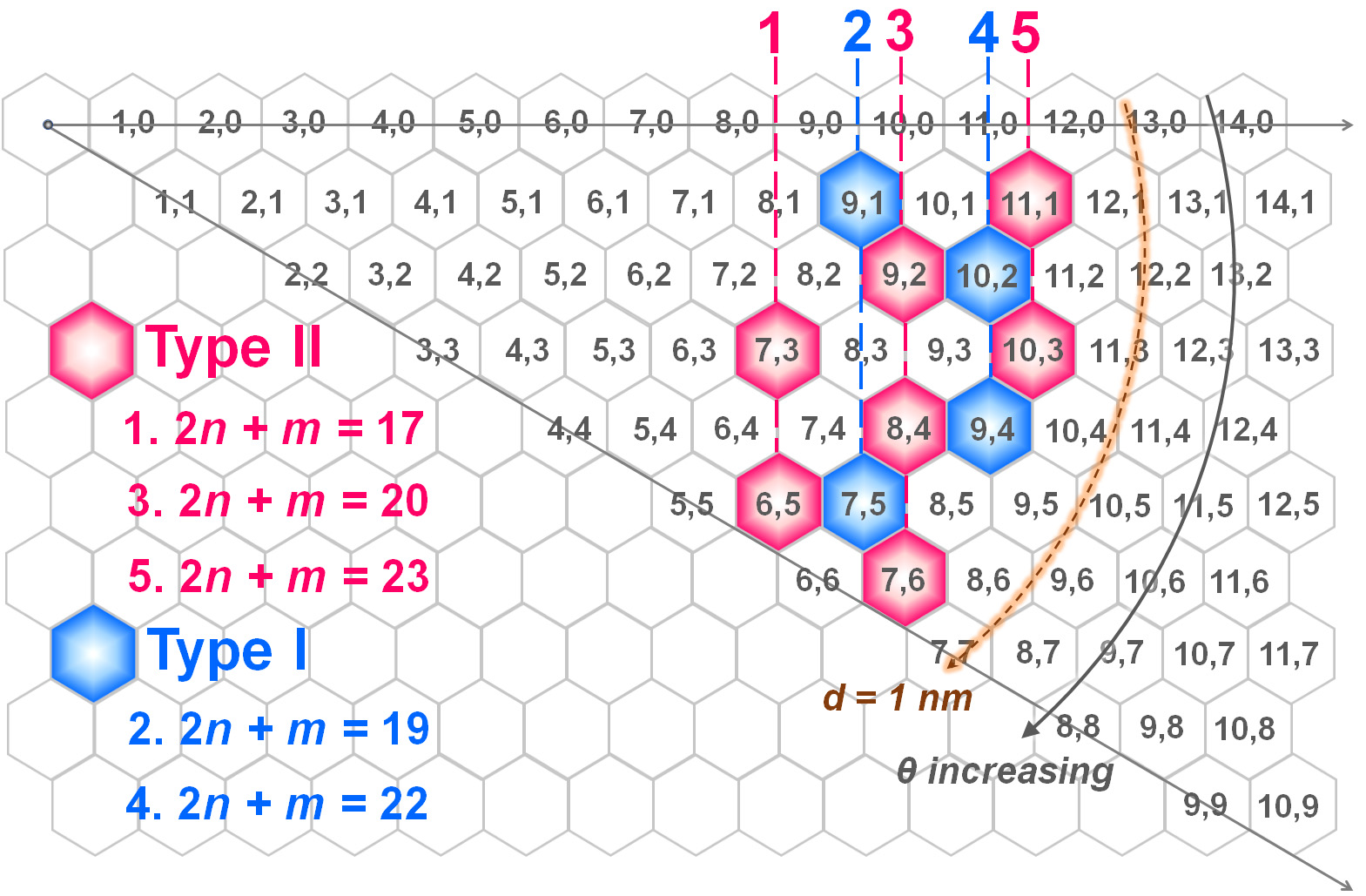

Supplement: Supplementary file 3 — Source Data [file 41467_2023_37443_MOESM3_ESM.zip › 7. Source Data/Figure 1/1c/SWCNT Type and family.jpg]

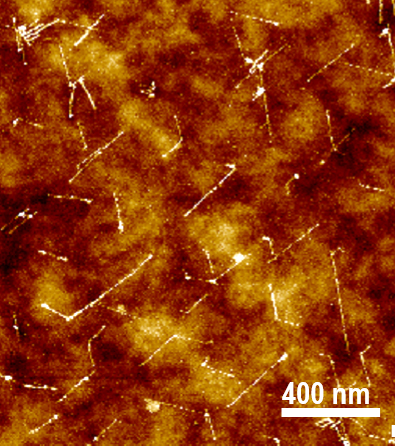

Supplement: Supplementary file 3 — Source Data [file 41467_2023_37443_MOESM3_ESM.zip › 7. Source Data/Figure 1/1d&e/AFM of SWCNTs.jpg]

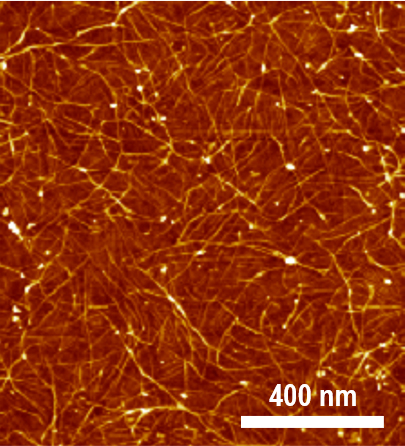

Supplement: Supplementary file 3 — Source Data [file 41467_2023_37443_MOESM3_ESM.zip › 7. Source Data/Figure 1/1f&g/AFM of SWCNTs.jpg]

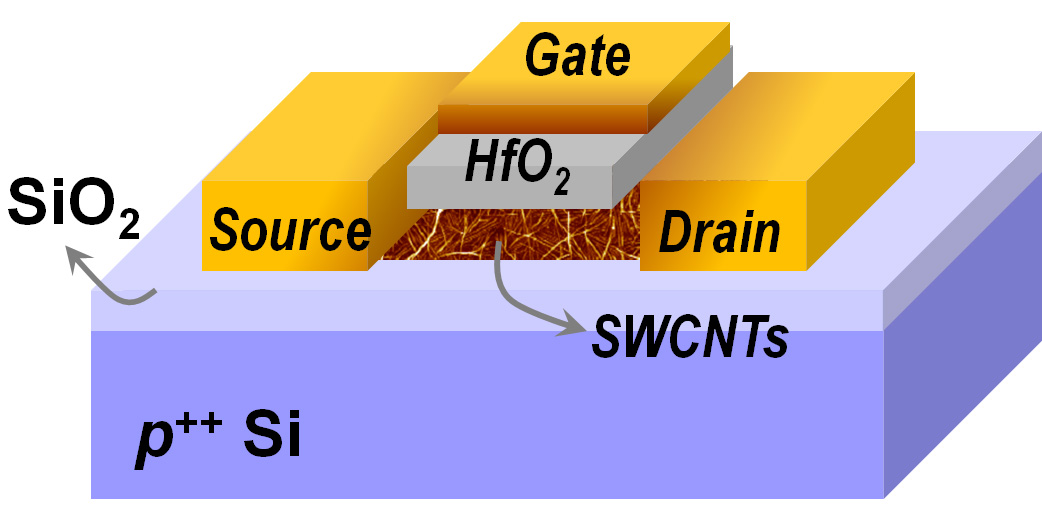

Supplement: Supplementary file 3 — Source Data [file 41467_2023_37443_MOESM3_ESM.zip › 7. Source Data/Figure 2/2a/2a.jpg]

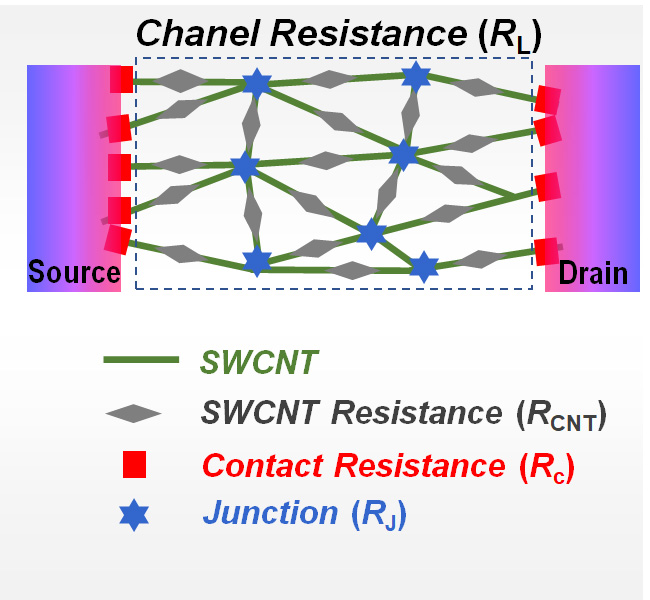

Supplement: Supplementary file 3 — Source Data [file 41467_2023_37443_MOESM3_ESM.zip › 7. Source Data/Figure 4/4a&b&c/4a.jpg]

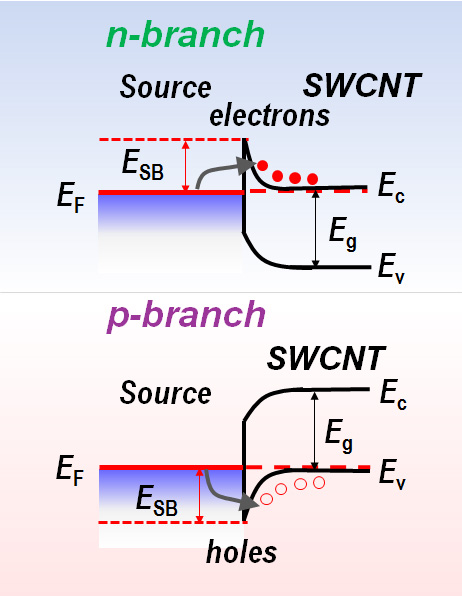

Supplement: Supplementary file 3 — Source Data [file 41467_2023_37443_MOESM3_ESM.zip › 7. Source Data/Figure 4/4a&b&c/4b.jpg]

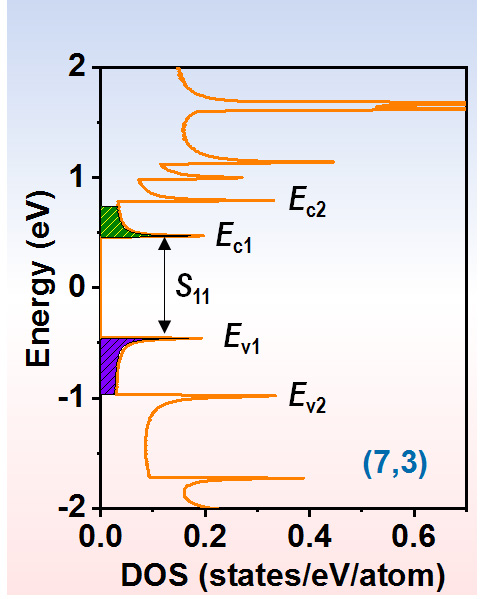

Supplement: Supplementary file 3 — Source Data [file 41467_2023_37443_MOESM3_ESM.zip › 7. Source Data/Figure 4/4a&b&c/4c.jpg]

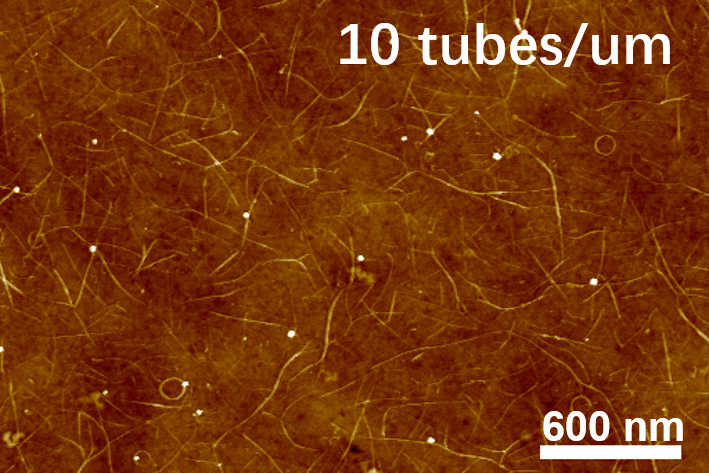

Supplement: Supplementary file 3 — Source Data [file 41467_2023_37443_MOESM3_ESM.zip › 7. Source Data/Figure S4/AFM of SWCNTs-10 tubes per um.jpg]

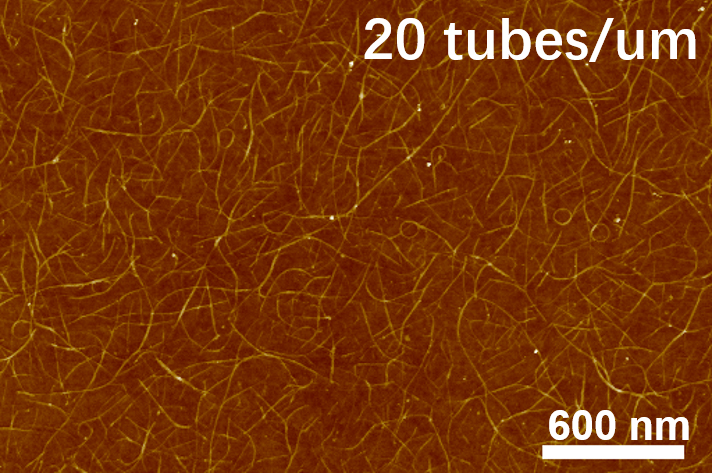

Supplement: Supplementary file 3 — Source Data [file 41467_2023_37443_MOESM3_ESM.zip › 7. Source Data/Figure S4/AFM of SWCNTs-20 tubes per um.jpg]

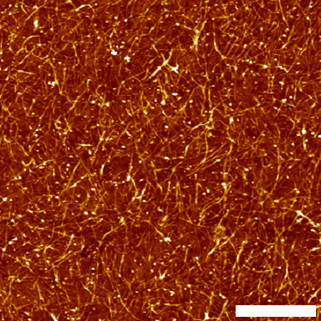

Supplement: Supplementary file 3 — Source Data [file 41467_2023_37443_MOESM3_ESM.zip › 7. Source Data/Figure S5/102.jpg]

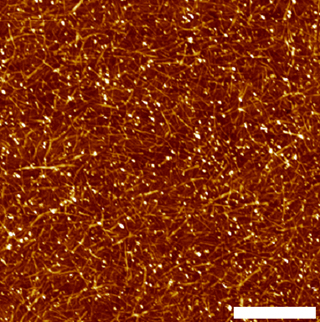

Supplement: Supplementary file 3 — Source Data [file 41467_2023_37443_MOESM3_ESM.zip › 7. Source Data/Figure S5/103.jpg]

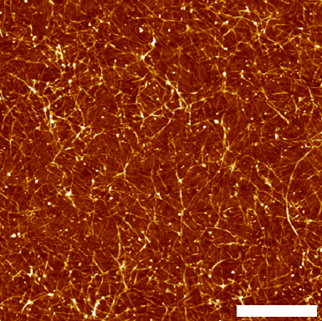

Supplement: Supplementary file 3 — Source Data [file 41467_2023_37443_MOESM3_ESM.zip › 7. Source Data/Figure S5/111.jpg]

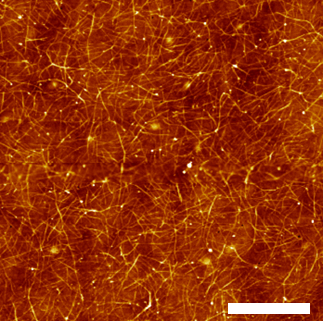

Supplement: Supplementary file 3 — Source Data [file 41467_2023_37443_MOESM3_ESM.zip › 7. Source Data/Figure S5/65.jpg]

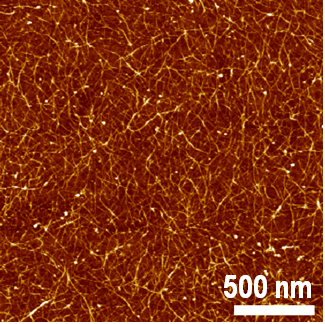

Supplement: Supplementary file 3 — Source Data [file 41467_2023_37443_MOESM3_ESM.zip › 7. Source Data/Figure S5/73.jpg]

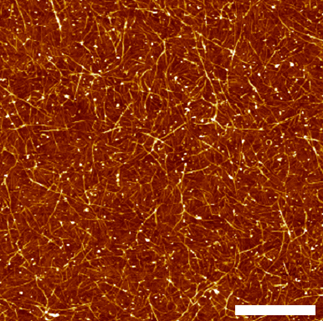

Supplement: Supplementary file 3 — Source Data [file 41467_2023_37443_MOESM3_ESM.zip › 7. Source Data/Figure S5/75.jpg]

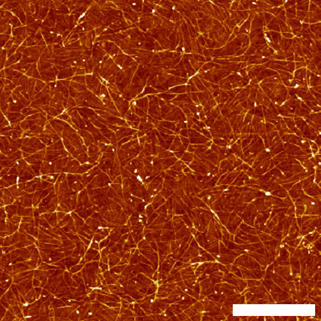

Supplement: Supplementary file 3 — Source Data [file 41467_2023_37443_MOESM3_ESM.zip › 7. Source Data/Figure S5/76.jpg]

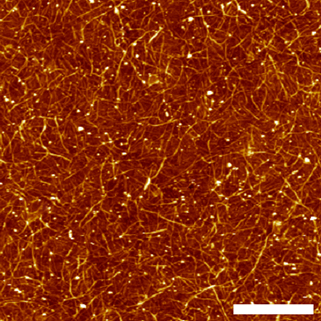

Supplement: Supplementary file 3 — Source Data [file 41467_2023_37443_MOESM3_ESM.zip › 7. Source Data/Figure S5/84.jpg]

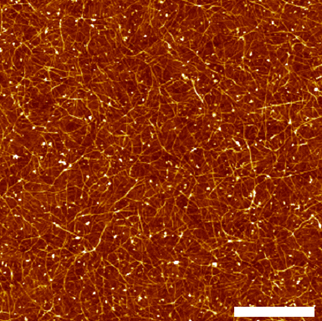

Supplement: Supplementary file 3 — Source Data [file 41467_2023_37443_MOESM3_ESM.zip › 7. Source Data/Figure S5/91.jpg]

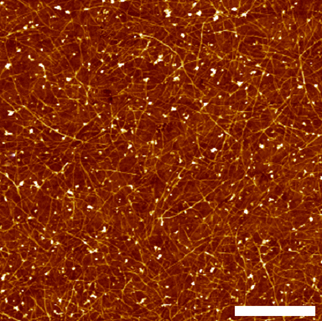

Supplement: Supplementary file 3 — Source Data [file 41467_2023_37443_MOESM3_ESM.zip › 7. Source Data/Figure S5/92.jpg]

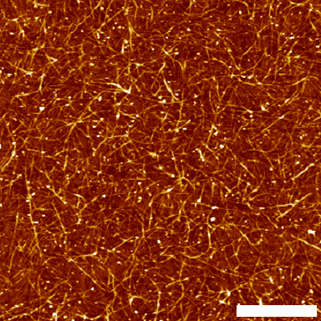

Supplement: Supplementary file 3 — Source Data [file 41467_2023_37443_MOESM3_ESM.zip › 7. Source Data/Figure S5/94.jpg]

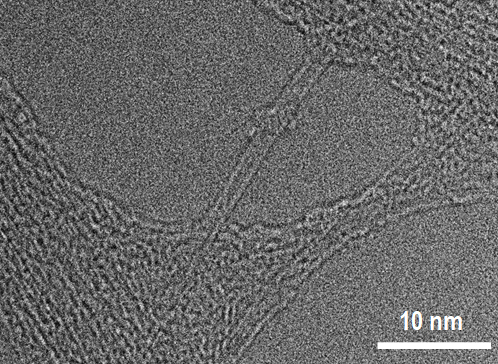

Supplement: Supplementary file 3 — Source Data [file 41467_2023_37443_MOESM3_ESM.zip › 7. Source Data/Figure S8/Figure S8-TEM.jpg]
